# Letter from Our Own Paris Correspondent

**Published:** 1888-01

**Authors:** 


### FOREIGN CORRESPONDENCE.

### LETTER FROM OUR PARIS CORRESPONDENT.

PARIS, 34 RUE DE BUCI, January 1, 1888.

*Editor Daniel's Texas Medical Journal:*

There are principally two questions, which furnish a great number of practical jokes in the amphi-theatres of the '*Ecole de Medicine*. They are the germ theory, and the admission to the study of medicine, of the female sex.

True, there is not so much infidelity on the subject of infection and antisepsis, in France, as there is in the United States, but it must be remembered, that the well known author and professor, M. Peter, is by no means an enthusiast on the subject of micro-organisms. It is easy to see that an authority of his rank would not fail to make an impression upon the young admiring student. The French student being on the whole a good deal more noisy and demonstrative than his American colleague, leaves no chance pass by without manifesting in some way or other his satisfaction, (or the opposite,) over the lectures.

It so happens that Prof. Blanchard is quite frequently interrupted, when he arrives at a decisive statement. Sometimes a sneering o–o–oh follows his sentences, other times a truly deafening applause rewards him. Of course, such frequent interruptions disturb. The professor will make a remark to that effect, but a regular thunder of noise follows. Some applaud, others stamp with their feet, or their umbrellas, others shout, others whistle, and a last party amuses himself with vociferations of an indescribable character. Soon comes a thundering "enough!" from all sides, but which is shouted with little sincerity, for it is rapidly followed by another volley of bravos. For five minutes everything seems disorder, then all is quiet again. If no second occasion shows itself, the last sentiments are saved for the end of the hour. Under a great applause, the professor leaves the pulpit, but he cannot help hearing the few single voices, which rend the air with the nick-names "micro-organism," "microgerm," etc.

While the admission of the female sex to the school of medicine, is by no means a recent innovation, the candidates of the fair sex are rather numerous this year. The "boys" of course are little pleased with their increasing attendance, and try all possible means of embarassing the poor girls. It is only necessary to make a start for a manifestation and everybody joins in, lecture or no lecture. Especially when a young lady comes in a little tardy, a demonstration is almost inevitable. A drawling "o-h" passing through every note for a nine scale piano comes from several hundred strong vocal apparatuses, and as the grand closing finale a general smacking of the lips, kissing and other indefinable sounds make themselves heard from every corner.

However, in the midst of all this jolliness, there is also earnest study, and with a faculty, as excellent as here, it is almost impossible not to learn anything. Our reader will be able to realize what an interest one must take in hearing such men as Prof. Duval Everything about him is originality. In his last lecture he stated that there was no more doubt as to the origin of the red blood-corpuscle. The places of their formation, are the spleen and the marrow of the bones. While in the oviparous animals the red cell is a simple transformation of a white blood globule, the fact differs for the viviparous animals, consequently for the human race. Neumann had pointed out, (1882,) that the white cell increases in size, and takes a red coloration from its taking up of hæmoglobin from the destroyed red disks. The red protoplasm now forms button like projections, *i. e.*, commences to bud, and those little buttons, detaching themselves, become new red blood disks. Hence, the latter are created by a process similar to the propagation by gemmation of the lower organisms.

In a recent lecture upon the subject of erysipelas, the great clinician of the Hospital de la Pitie, Prof. Jaccoud, made several remarks, which we think well worthy of a reproduction :

"As a premonitary symptom, vomiting is not frequent. Contrary to the general statements, its absence is the rule. In the same way, the swelling of the ganglia is by far more rare, than has been asserted. In the absence of cerebral symptoms, the temperature rarely exceeds 103–104°, even if the scalp be invaded. The average duration of simple erysipelas, is 6–10 days. Such cases are almost never followed by a fatal issue. As a rule, they terminate by crisis—*a distinct sign that the infection is of infectious origin.* A crisis may be prognosticated in all cases, where the diseases proceeds in exacerbations, (*par poussees.*) Among the complications of particular interest are the inflammations of the mitral valve. (endocarditis,) manifesting themselves by a slight systolic murmur before or during the onset of the malady. In those cases, a spontaneous cure follows in 90 per cent. Another frequent complication is nephritis. DaCosta has maintained its invariable presence. Clinical observation has shown this to be wrong. Even albumimia, not due to inflammatory condition of the kidney, is often absent.

The intestines can aggravate the trouble, by a simple catarrh occupying as a rule, only the duodenum. A more serious form is an intestinal ulceration. The ulcers hardly ever exceed five in number, implicate only the mucous membrane and usually occur in the small intestines. In such a case the eruption may be ushered in by repeated bilious vomiting, epigastric pain, tenesmus and dysenteric dejecta."

Knowing how disagreeable the treatment of chronic articular rheumatism becomes to the practitioner in the absence of any visible improvement, even after a prolonged course of remedies, I believe, that a short synopsis of the treatment, as given by the "big guns" in Paris would be appreciated. The well known author and director of the medical division of the "Hospital de la Charite," Prof. Potain, gives the following directions :

The treatment is hygienic and medical. The former implies avoiding all exciting causes, good nourishment and moderate exercise. The medical treatment differs during the apyretic periods and the febrile attacks. In the latter case he administers the salts of quinine, antipyrine, or sodium salicylate. The latter is by far less efficacious, than in the acute form. During the apyretic periods the internal treatment consists of the administration of potassium iodide three times a day. The daily quantity may be pushed to 40–50 gr. In anæmic and weak persons, arsenic in form of Fowler's solution is preferable. The external measures comprise baths and electricity. The water should be as much as possible of the same temperature. The room must be heated and great care be exercised to prevent refrigeration of the patient. The baths should last from 1–2 hours. In severe cases, the water of

Aix and sulphurous douches are to be employed. As electrical remedy the continued current ought to be made to traverse the water in which the patient is bathing.

A new factor in the genesis of epilepsy, was discussed in the last meeting of the Academy of Sciences. It is the condition known as otopiesis, which means a rarefaction of air in the tympanic cavity. When the eustachian tubes are occluded, (inflammatory swelling, mucous, foreign body, etc.,) the quantities of air undergoing gradual absorption in the middle ear, cannot be replaced. From this results negative pressure able to excite a reflex epilepsy. In all those cases the patient will complain of a considerable degree of deafness,—a sign which always should attract attention. The fit is then usually preceded by noises in the ear of the most divers character. The trouble is at once arrested by inflation, and treating such causative factors as pharyngitis. We should, therefore, always convince ourselves of the permeability of the eustacian tubes.

A truly utopian report on the action of antipyrine in sea sickness, was communicated by Dr. Dupuy. Selecting the persons most liable to the affection, (dyspeptics especially, with dilation of the stomach,) he gave them 45 grains of antipyrine daily, so as to commence three days before the voyage. He discontinued the treatment after six days, and had the pleasure of seeing that not a single person under treatment was attacked.

M. Pasteur has also been making the rounds in the daily press since a few weeks. With a truly American spirit as to the practical question, he advises the application of the chicken cholera microgerm for the destruction of the rabbits, which constitute a veritable plague in some countries. He has proven, that the innoculation is very easy. A few rabbits, once affected, they would soon spread the epidemic to a complete destruction of their tribe. No other domestic animals, but the chickens, can take the disease, and M. Pasteur thinks that a "chicken quarantine" could easily be effected. The French papers are wondering how such a marvellous idea could be conceived. *Ah, oui, toujours le illustre Savant.*

